# Knowledge and attitudes of midwives on the use of the partogram: a study among midwives in the Tamale Metropolis

**DOI:** 10.1186/s40748-016-0030-0

**Published:** 2016-04-25

**Authors:** Kennedy Diema Konlan, Joseph M. Kombat, Mary Gifty Wuffele, Milicent Aarah-Bapuah

**Affiliations:** Tamale Teaching Hospital, Tamale, Ghana; Department of Paediatrics, University for Development Studies, School of Allied Health Sciences, Tamale, Ghana; Department of Midwifery, University for Development Studies, School of Allied Health Sciences, Tamale, Ghana; Department of Nursing, University for Development Studies, School of Allied Health Sciences, Tamale, Ghana

**Keywords:** Partogram: a graphical tool that is used to assess the progress of labour and to identify when interventions are necessary, Labour: the processes in a woman (37 weeks gestation) for successful expulsion of the product of conception, Maternal complications: any untoward event, scenario or process that occurs to the mother with before, during and immediately after birth

## Abstract

**Background:**

The partogram is the most effective tool to use to monitor the progress of labour and complications associated with labour.

The main objective of this study was to assess the knowledge level of midwives on the effective use of the partogram in monitoring the progress of labour in the Tamale Metropolis of Ghana.

**Methods:**

This study was a cross-sectional descriptive study conducted on 140 midwives selected using random sampling technique from March to July, 2011.

**Results:**

The study revealed that all respondents had knowledge of the supposed use of the partogram. However, the study also identified inadequate knowledge on the proper use of the tool (as observation showed that some of the partogram sheets were inadequately filled). Inadequate knowledge, inadequate staffing (1.0 % of respondents) and extra workload on the few midwives (2.6 %) were some of the factors that militated against the effective use of the partogram. There were enough partogram sheets in the health facilities under study as 91.4 % of respondents had access to the partogram.

**Conclusion:**

Midwives in Ghana know about the supposed use of partogram as a monitoring tool for labour; however inadequate knowledge on the proper use of the tool and inadequate staffing militated against effective use. We recommended constant refresher training on the use of the partogram and also training of more midwives to enhance effective utilisation of the partogram.

## Background

A considerable number of women suffer complications from labour and some of these complications result in maternal and infant mortality. According to recent global estimates, about 289, 000 women die annually from pregnancy related complication [1]. Most of these deaths occur in the developing world. Ghana like other developing countries has a high maternal mortality rate. The Ghana Maternal Health Survey 2007 indicates that maternal mortality ratio in Ghana remains unacceptably high at 451 deaths per 100,000 live births.

The partogram is a sigmoid curve tool that can be used to assess the progress of labour and to identify when intervention is necessary: It is a graphical record of cervical dilatation in centimetres against duration of labour in hours. Studies have shown that using the partogram can be highly effective in reducing complications from prolonged labour for the mother (postpartum haemorrhage, sepsis, uterine rupture and its sequelae) and for the new-born (death, anoxia, infections among others), [2]. In addition to increased morbidity and mortality for women, improper usage of the partogram can lead to an increase in unnecessary interventions [3].

The United Nations noted that the use of the Partogram ensured that, there is a comprehensive monitoring of the progress of labour as well as maternal and foetal wellbeing. This makes it possible to address in a timely manner the complications that arise [4]. Using the Partogram can be highly effective in reducing complications from prolonged labour for the mother and for the new born [5]. The introduction of the partogram also led to the reduction in augmentation and Caesarean section rate [2, 6].

Ghana adopted the use of the WHO Partogram in labour wards throughout the country in 1990 with the intention of improving maternal health and reducing maternal and perinatal mortality [7]. This was probably part of quality assurance measures on the labour units [8].

Although a number of studies have been conducted in the area of maternal and infant mortality, leading to the implementation of several interventions aimed at reducing the trends, several maternal and perinatal mortalities still occur as a result of obstructed labour. It is in this regard that, it is important to collect relevant data on knowledge and attitudes of midwives towards the effective use of the partogram. This study also sought to find out the factors that influence the effective use of the Partogram by midwives. It also provided a better understanding on the importance of using the Partogram effectively to monitor labour.

### Objective of study

The goal of this study was to assess the knowledge level and attitudes of midwives towards the effective use of the partogram in monitoring the progress of labour in Tamale Metropolis.

## Methods

### Study design

The study design adopted is a descriptive study design that assessed the level of knowledge and implementation on the use of the partogram by midwives. The specific study design adopted was cross-sectional study.

### Population and sample

The total number of midwives within the Tamale Metropolis was two hundred and fifteen (215). The study was done among one hundred and forty midwives from three main hospitals in the Metropolis. The hospitals included; Tamale Teaching hospital, West Hospital, and Tamale Central hospital. Midwives were chosen because they are those who monitor labour and conduct deliveries for pregnant women in the hospitals. The partogram is a tool that helps them to assess whether labour is progressing normally or not, so that any necessary intervention could be taken.

A sample of one hundred and forty midwives was selected using random sampling technique with a 95 % confidence interval and 5 % reliability. There was one hundred percent response rate to the questionnaire. Sample size was calculated using the formula by Glenn D. Israel.

### Data collection methods

The study was mainly conducted using a structured questionnaire, however, interviews and direct observation of respondents provided validation of the data. A structured questionnaire was designed and administered to each respondent. The questionnaire included questions on socio-demographic characteristics, knowledge on the partogram, factors that influence the effective use of the partogram, availability of the partogram in the health facilities and number of deliveries conducted using the partogram.

### Data processing and analysis

Data collected was entered into Microsoft Excel 2007 spread sheet and imported to SPSS (Statistical Package for Social Sciences) version 16 for analysis. Results were presented with simple statistical tools.

### Pretesting

In order to test for the validity and reliability of the research’s data collection methods, a pre-test of the questionnaire was carried out among five midwives of the Bolgatanga Municipal hospital. Their responses with regard to the questions asked enabled the researcher to re-phrase the questions’ sentences/language to enhance clarity and precision.

### Ethical considerations

Introductory letters with information sheets of the study was submitted to the Research Unit of the Tamale Teaching hospital, West and Central hospitals following which permission was granted for the study to be conducted.

## Results

This study included midwives within the Tamale Teaching Hospital, West Hospital and Central Hospital. A total of one hundred and forty (140) midwives completed the research questionnaire. The results of the study indicate that, the majority of the midwives (54.3 %) were between the ages of 51–60 years whiles the least number of respondents were those between 20 and 30 years (8.6 %). The number of respondents who have been working for the past 21–30 years were 30, representing 21.4 % and those who have worked for 31 years and above were 14 (10.0 %). It was revealed that a considerable number of midwives (53.6 %) have access to in-service training on the use of the partogram once every six months whiles 20.7 % do so once in a year. However, 19 midwives out of the 140 (13.6 %) had not gone for in-service training during the period of their practice.

Findings on the knowledge of midwives about the use of the partogram, indicated that all respondents have heard about the tool. Majority (76.4 %) further indicated that the partogram is a tool used to monitor the progress of labour while 15.7 % indicated that the partogram could both be used to monitor labour and to check maternal and foetal conditions. In addition to what was already mentioned, 7.9 % added that it could also be used to check for any abnormalities in the foetus. This study revealed that only 40.0 % of midwives responded correctly when they were to determine when to start plotting on the partogram. The remaining 60.0 % had varied responses that were not consistent with the time to start plotting on the partogram.

An analysis of the knowledge of midwives about symbols provided on the questionnaire of this study about cervical dilatation indicated that 95.7 % of the respondents agreed that the symbol **X** was used in contrast to 4.3 % who were of the view that it was plotted with the symbol **O.** Also, 92.9 % of the respondents demonstrated that, descent of the foetal head was plotted using the symbol **O,** whereas 7.1 % did not know what symbol was used.

There was varied access to the partogram as 91.4 % of the midwives had access to the partogram. Careful observational method used during the study identified that some of the partogram sheets were inadequately or improperly filled. The 8.6 % of the midwives that answered no to the usage of the partogram in their health facilities gave the following reasons for their response: unavailability of the partogram sheets (3.4 %), inadequate number of staff (1.0 %), too much workload on midwives (2.8 %), and inadequate knowledge on the use of the partogram (1.4 %). Majority (72.1 %) of the midwives acknowledged, there were enough partogram sheets in their facilities ranging between 21 and 500 sheets.

## Discussion

The age distribution of midwives in the Tamale metropolis support the view that most of the midwives are, ageing and will soon retire especially when they approach sixty (60) years which is the retirement age for service workers in Ghana. This age distribution also means that, some midwives were trained before the implementation of the partogram as an international standard tool and are most likely not to have adequate knowledge about the partogram. Until this declaration, training of midwives did not mandatorily incorporate the use of the partogram. This necessitates continues in-service training of midwives on the use of the partogram as a refresher means for effective implementation of the tool in health facilities. Without this in-service training as espoused by some midwives, the use of the partogram in health facilities is bound to have some challenges.

African reproductive health journal reported in 2008 that, the partogram is made up of the following components: progress of labour, foetal condition and maternal condition. It is therefore a tool that is used to monitor these parameters during labour [9]. Midwives in this study demonstrated inadequate knowledge on the proper use of the partogram as a labour monitoring tool since a minute proportion were able to identify all the three components of the use of the partogram. Midwives’ inadequate knowledge on the proper use of the partogram is consistent with findings of a study that was conducted among some health facilities in Rujumbura sub-district in Rukungiri district of Uganda, where out of 1674 deliveries across all the health units only 735 of the cervical dilation and 396 uterine contractions were plotted on the partograms to recommended standard [10].

Most midwives in this study could not identify the specific time to commence recording on the partogram. According to the journal of the African Reproductive Health, the active phase of labour begins when the cervix is 4 cm dilated, a point where plotting on the partogram should begin. On the World Health Organisation (WHO) partogram, cervical dilatation is measured by the diameter in centimetres. This is recorded with an **X** in the centre of the partogram, at the intersection of the horizontal line whiles descent of the foetal head is measured by abdominal palpation and expressed in number of finger widths (Fifths of the head) above the pelvic brim. It is recorded in the central part of the partogram with an **O**. The specific identification of these symbols is important for smooth communication among the health care team members. However, most of the midwives included in this study could not identify the recordings on the partogram according to this guidelines stipulated by the WHO. Based on this reasons of not effectively using the tool as well as the inability to plot readings on the partogram, it can be deduced that the partogram is not effectively used in most health facilities in the Metropolis as reported in similar studies This study also identified three factors that influenced the utilisation of the partogram; non availability of the sheets, time consuming nature of the partogram and inadequate staffing. These findings agreed to the results of the study in Kenya where staff shortages, lack of knowledge especially on interpretation of findings, negative attitudes, conflict between providers as to their roles in filling the partogram, were among factors that contributed to poor usage of the partogram [11].

A study in Tanzania (*n* = 196), concluded that although the partogram was available for most eligible deliveries in three hospitals studied, ‘the overall’ proportion of implementation was only 58 % [12]. The results of this study pinpointed the fact that the partogram sheets were used daily depending on the number of deliveries worth using them (partogram sheets). A minimum of between 1 to 5 and a maximum of 40 partogram sheets were used per day in each facility.

Responses given by interviewed midwives concerning the benefits derived from the use of the partogram during labour, coupled with other suggestions they made as to how to ensure the effective use of the partograam in all health facilities in the Metropolis, provided enough proof that they were fully aware of its importance. With 91.4 % of midwives having access to the partogram, makes it cost effective.

## Conclusions

Midwives in the Tamale Metropolis generally have high access to partogram sheets as was revealed in the study that access to the partogram did not pose as a challenge in using it. However the results of the study further revealed an inadequate knowledge on how to plot information on the partogram, particularly the symbols used. Generally the study showed ineffective usage of the partogram by midwives within the metropolis.

### Recommendations

Based on the findings of the study we recommend training and employment of more midwives and continuous in-service training on the use of partogram as well as constant monitoring and supervision of health professionals particularly midwives to ensure effective use of the partogram.

## Abbreviations

CHEW: Community health extension workers
Cm: Centimetres
CPD: Cephalo pelvic disproportion
FMC: Federal medical centre
GSS: Ghana statistical service
MNH: Maternal and neonatal health programme
MOH: Ministry of health
NDUTH: Niger delta university teaching hospital
SPSS: Statistical package for social sciences
UNFPA: United nations population fund
UNICEF: United nations children fund
WHO: World Health Organization
